# Isolation and Characterization of Carbendazim-degrading *Rhodococcus erythropolis* djl-11

**DOI:** 10.1371/journal.pone.0074810

**Published:** 2013-10-01

**Authors:** Xinjian Zhang, Yujie Huang, Paul R. Harvey, Hongmei Li, Yan Ren, Jishun Li, Jianing Wang, Hetong Yang

**Affiliations:** 1 Shandong Provincial Key Laboratory of Applied Microbiology, Biotechnology Center of Shandong Academy of Sciences, Jinan, Shandong Province, People's Republic of China; 2 CSIRO Sustainable Agriculture National Research Flagship and CSIRO Ecosystem Sciences, Glen Osmond, South Australia, Australia; University of Kansas, United States of America

## Abstract

Carbendazim (methyl *1H*-benzimidazol-2-yl carbamate) is one of the most widely used fungicides in agriculture worldwide, but has been reported to have adverse effects on animal health and ecosystem function. A highly efficient carbendazim-degrading bacterium (strain dj1-11) was isolated from carbendazim-contaminated soil samples via enrichment culture. Strain dj1-11 was identified as *Rhodococcus erythropolis* based on morphological, physiological and biochemical characters, including sequence analysis of the 16S rRNA gene. *In vitro* degradation of carbendazim (1000 mg·L^−1^) by dj1-11 in minimal salts medium (MSM) was highly efficient, and with an average degradation rate of 333.33 mg·L^−1^·d^−1^ at 28°C. The optimal temperature range for carbendazim degradation by dj1-11 in MSM was 25–30°C. Whilst strain dj1-11 was capable of metabolizing cabendazim as the sole source of carbon and nitrogen, degradation was significantly (P<0.05) increased by addition of 12.5 mM NH_4_NO_3_. Changes in MSM pH (4–9), substitution of NH_4_NO_3_ with organic substrates as N and C sources or replacing Mg^2+^ with Mn^2+^, Zn^2+^ or Fe^2+^ did not significantly affect carbendazim degradation by dj1-11. During the degradation process, liquid chromatography-mass spectrometry (LC-MS) detected the metabolites 2-aminobenzimidazole and 2-hydroxybenzimidazole. A putative carbendazim-hydrolyzing esterase gene was cloned from chromosomal DNA of djl-11 and showed 99% sequence homology to the *mheI* carbendazim-hydrolyzing esterase gene from *Nocardioides* sp. SG-4G.

## Introduction

Carbendazim (methyl *1H*-benzimidazol-2-yl carbamate, MBC) is a systemic benzimidazole fungicide widely used in many countries to control a broad range of fungal diseases of agricultural crops [1]. MBC is the hydrolytic product and active component of some other widely used benzimidzaole fungicides such as benomyl and thiophanate methyl [2], [3]. MBC is relatively stable in soil and water and is reported to have an environmental half-life of up to 12 months [4]. The soil persistence and the plant systemic nature of MBC can in turn, lead to the contamination of water and plant products [5]. This causes serious concerns because MBC is a suspected mutagen, teratogen and carcinogen and is reported to be toxic to mammalian liver, endocrine and reproductive tissues [6], [7]. Residual MBC in soil has also been reported to alter the taxonomic structure of soil bacterial communities and may therefore adversely affect microbial-mediated ecosystem functions [8].

There is an increasing demand to remediate soils contaminated with MBC because of the prolonged use of the fungicide in agriculture, its environmental persistence and adverse impacts on animal health. Degradation rates of MBC by physical and abiotic chemical processes are reported to be slow, with microbial metabolism thought to be the principal degradative process in natural soils [4], [9], [10]. Only a limited number of MBC-degrading bacterial strains have been previously reported [4], [5], [11] and highly efficacious, ecologically competitive microbes are required to remediate a range of MBC contaminated environments. A gene-enzyme system for MBC degradation has been previously reported in *Nocardioides* sp. [4], but mechanisms utilized by other MBC-degrading microbes are yet to be elucidated. In this study, we describe the isolation of a highly efficacious MBC-degrading *Rhodococcus erythropolis* strain djl-11, conditions affecting MBC bio-degradation by this strain and sequence characterization of the dj1-11 MBC-hydrolyzing esterase gene.

## Materials and Methods

### Chemicals and growth media

Analytical-grade carbendazim (MBC), 2-aminobenzimidazole (2-AB) and 2-hydroxybenzimidazole (2-HB) were purchased from Sigma-Aldrich Inc. All other chemicals and solvents were of highest analytical-reagent grade.

Liquid minimal salts medium (MSM) consisted of 1.0 g NH_4_NO_3_, 1.0 g NaCl, 1.5 g K_2_HPO_4_, 0.5 g KH_2_PO_4_, 0.2 g MgSO_4_·7H_2_O per liter. Unless otherwise stated, MSM was adjusted to pH 7.0 and MBC was added at a final concentration of 1000 mg·L^−1^ in powdered form. Solid carbendazim- amended MSM contained 15 g agar L^−1^, the carbendazim solution added to the cooled medium after autoclaving. Luria-Bertani (LB) medium was used for general bacterial growth.

### Isolation of MBC degrading microorganisms

Soil samples were taken from vineyards in Rizhao (Shandong Province, China) with a 10-year history of repeated MBC applications. To select for MBC-degrading microbes, 10 g of soil was placed in a 500 mL Erlenmeyer flask containing 100 mL of MSM supplemented with 1000 mg·L^−1^ MBC (*i.e.* MSM-C_1000_) as the sole carbon source and incubated at 28°C on a rotary shaker (150 rpm). After 7 days, 5 mL of culture was inoculated to 100 mL fresh MSM-C_1000_ and incubated under the same conditions for another 7 days. After 5 sequential rounds of enrichment (*i.e.* 35 days exposure to MSM-C_1000_), 100 μL of culture was plated onto MSM-C_1000_ agar and incubated at 28°C for 5 days. Colonies showing transparent halos indicative of MBC-degradation were streaked onto fresh MSM-C_1000_ agar plates to confirm MBC degradation [4] and single cell colonies were purified for further analyses.

### Identification of MBC-degrading bacteria

Identification of MBC-degrading bacteria was based on morphological, physiological and biochemical characterization according to Bergey's Manual of Systematic Bacteriology [12]. Molecular taxonomy was based on PCR amplification and DNA sequencing of the 16S rRNA gene with the universal primers 8F (5′-AGAGTTTGATCCTGGCTCAG-3′) and 1541R (5′-AAGGAGGTGATCCAGCCGCA-3′) according to established protocols [13]. PCR primer synthesis and DNA sequencing (Applied Biosystems) were conducted at Sangon Biotech Co. Ltd., (Shanghai, China). The resulting nucleotide sequences were compared to those in GenBank using a BLAST search.

### Microbial MBC degradation

MBC biodegradation was quantified by monitoring decreasing concentrations of the fungicide in liquid culture over time. Strain dj1-11 was grown in LB broth at 28°C on a rotary shaker (150 rpm) for 24 h. Cells were collected by centrifugation (6000 g for 5 min.), washed twice and re-suspended to an OD_600_ = 0.8 (Lambda Bio Spectrophotometer, Perkin Elmer, USA) in sterile water. The cell suspension (approx. 1×10^8^ cells·mL^−1^) was used to inoculate (1% v/v) 100 mL flasks of MSM-C_1000_ and incubated at 28°C on a rotary shaker (150 rpm). Uninoculated MSM-C_1000_ served as the negative control and each treatment was replicated 3 times. Culture samples were collected at 6 h intervals over a 72 h growth period, and 4 volumes of acetone were added to each sample. Samples were mixed well and stored at 4°C until analyzed by liquid chromatography (LC) and LC-mass spectrometry (LC-MS).

To study the effects of MBC concentration on degradation by dj1-11, cell suspensions were prepared as described above, inoculated (1% v/v) to 100 mL of MSM containing 200, 400, 600, 800 and 1000 mg·L^−1^ MBC and incubated at 28°C on a rotary shaker (150 rpm) for 48 h. Uninoculated flasks of MSM comprising the 5 MBC concentrations served as the negative controls. Each treatment was replicated 3 times. All samples were collected and prepared as described above for subsequent LC and LC-MS analyses.

### Identification and quantification of MBC and its metabolites

Quantitative analysis of MBC, 2-AB, and 2-HB was conducted with an Agilent series LC system (Agilent Technologies, USA). Chromatographic separation was achieved on an Eclipse XDB-C18 column (150 mm×4.6 mm, 5-μm particle size) at 25°C. MBC and its metabolites were monitored at 270 nm using an acetonitrile-water mixture (16∶84 [v/v] containing 0.1% [v/v] formic acid) as a mobile phase, at a flow rate of 1 mL·min^−1^. Metabolites were qualitatively analyzed by a LC-MS mass spectrometer (Agilent Technologies, USA). The separated substrate and metabolites were ionized with positive polarity and scanned within a mass range of 29 to 500 m/z.

### Effects of organic substrates, bivalent cations, pH and temperature on MBC degradation

Four experiments were established to determine the individual effects of organic substrates (N and C sources), cations, pH and temperature on MBC degradation by dj1-11. Unless otherwise indicated, bacterial inoculum was prepared, cultured in MSM-C_1000_ (48 h) and MBC degradation analyzed using the methods described above. Each treatment of the 4 experiments was replicated 3 times.

To examine the effects of organic substrates as alternative sources of nitrogen and supplementary sources of carbon on MBC biodegradation, 0.1% peptone, 0.1% beef extract, 0.1% urea, and 0.1% yeast extract (w/v) were respectively added to MSM-C_1000_ in the absence of NH_4_NO_3_. MSM with no nitrogen source was included as the negative control. Similarly, substitutions of the 810.8 μM MgSO_4_ in MSM-C_1000_ with equimolar amounts of ZnSO_4_, MnSO_4_, CuSO_4_, CaSO_4_ or FeSO_4_ were used to determine the effects of alternative bivalent cations on MBC biodegradation.

The effect of initial MSM-C_1000_ pH on MBC biodegradation was observed on a scale of pH 4 to pH 9 at increments of 1 pH unit. Optimal culture incubation temperatures for biodegradation of MBC were examined using 5°C increments on a scale of 20°C to 40°C.

### Cloning of the MBC-hydrolyzing esterase gene

A putative MBC-hydrolyzing esterase gene was amplified by PCR from chromosomal DNA of djl-11. The primers MheI-F (5′-gcatggccaacttcgtcctcg-3′) and MheI-R (5′-gcgcccagcgccgccagc-3′) were designed according to the sequence of a MBC-hydrolyzing esterase encoding gene *mheI* (GenBank accession GQ454794) [4]. For PCR amplification, approximately 270 ng of djl-11 gDNA, 5 μL of 10× PCR buffer (Mg^2+^ free), 3 μL of 25 mM MgCl_2_, 4 μL of 2.5 mM dNTPs, 20 pmol of each primer, and 1.25 U of TaKaRa Taq polymerase (TaKaRa Bio Inc., Dalian) were added to a final volume of 50 μL. PCR amplification was carried out as follows: 4 min at 95°C, 30 cycles of 40 sec at 95°C, 30 sec at 58°C and 40 sec at 72°C, plus a final extension step of 8 min at 72°C. The amplified product was purified using an Agarose gel DNA purification kit (TaKaRa Bio Inc., Dalian), inserted into the T-A cloning vector pMD18-T (TaKaRa Bio Inc., Dalian) and sequenced on an Applied Biosystems DNA analyzer. PCR primer synthesis and DNA sequencing were conducted at Sangon Biotech Co. Ltd. (Shanghai, China). The nucleotide sequence was compared to those in GenBank using a BLAST search.

### Statistical analysis

Biodegradation of MBC by strain dj1-11 was assessed by comparing differences in MBC concentration between treatments, each consisting of 3 replicates. All data were analyzed by analyses of variance (ANOVA), using SPSS 16.0 statistical software (SPSS Inc., USA). Pairwise comparisons of means were used to compute Fisher's least significant difference values (LSD, P = 0.05).

### Ethics statement

We confirm that the owner of vineyards gave permissions to take soil samples from the fields. We confirm that no endangered or protected species were involved in field studies.

## Results

### Isolation and identification of MBC-degrading strain dj1-11

Enrichment cultures established from MBC-contaminated soils were plated onto MSM-C_1000_ agar to select for putative MBC degrading microbes. Bacteria representing different colony morphologies were purified and confirmed to have MBC degradative function via plate-clearing assays. Strain dj1-11, qualitatively assessed as the most effective MBC degrader, was selected for further study.

Strain djl-11 was a gram-positive, non-motile, rod-shaped bacterium that formed orange colonies on LB agar after 72 h at 28°C. In physiological and biochemical tests, dj1-11 tested positive for catalase, urease and acetoin produciton, but negative for oxidase, starch hydrolysis and nitrate reductase. Strain dj1-11 was able to utilise citrate, mannose, sodium benzoate and maltose as sole carbon sources and acetylamine and asparagine as sole sources of carbon and nitrogen.

**Figure 1:**
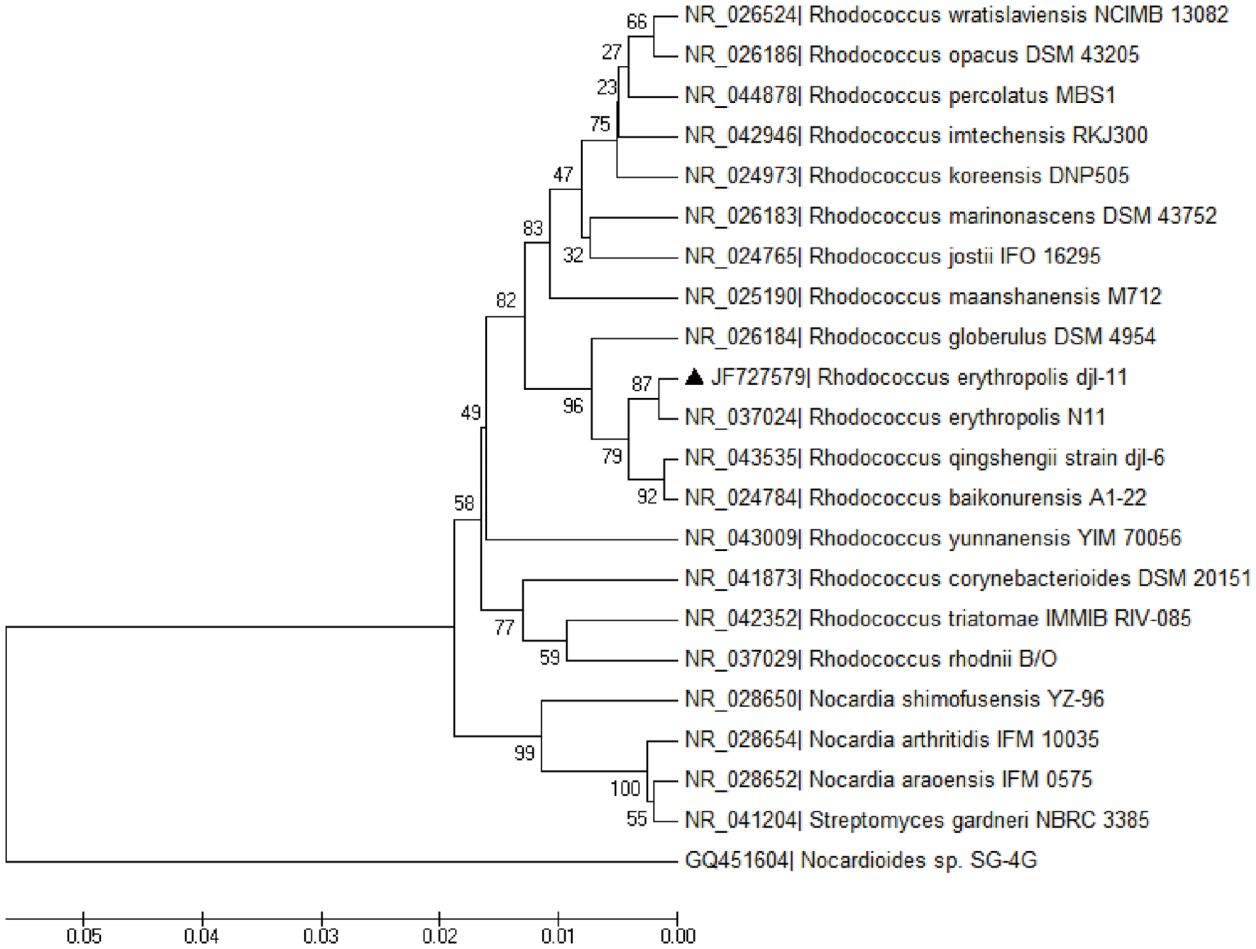
Phylogenetic tree based on the 16S rDNA sequence of strain djl-11 and related species. Strain djl-11 is marked with “▴”. The percentages of replicate trees in which the associated species clustered together in the bootstrap test (1000 replicates) are shown next to the branches. Genbank accession numbers are shown.

A 1.5 kb 16S rRNA fragment was amplified from strain djl-11, sequenced and showed 99% homology to *Rhodococcus erythropolis* 16S rRNA. Phylogenetic analysis (Figure 1) based on 16S rDNA sequences revealed that strain djl-11 clustered with *Rhodococcus* species and was most closely related to *R. erythropolis* N11. Molecular taxonomy, cellular and colony morphologies and physiological and biochemical characteristics identified djl-11 as *R. erythropolis*. Strain djl-11 and its 16s rRNA sequence were deposited in the China General Microbiological Culture Collection Center (Accession No. CGMCC4554) and GenBank (Accession No. JF727579), respectively.

### MBC biodegradation and metabolite identification in liquid culture

**Figure 2:**
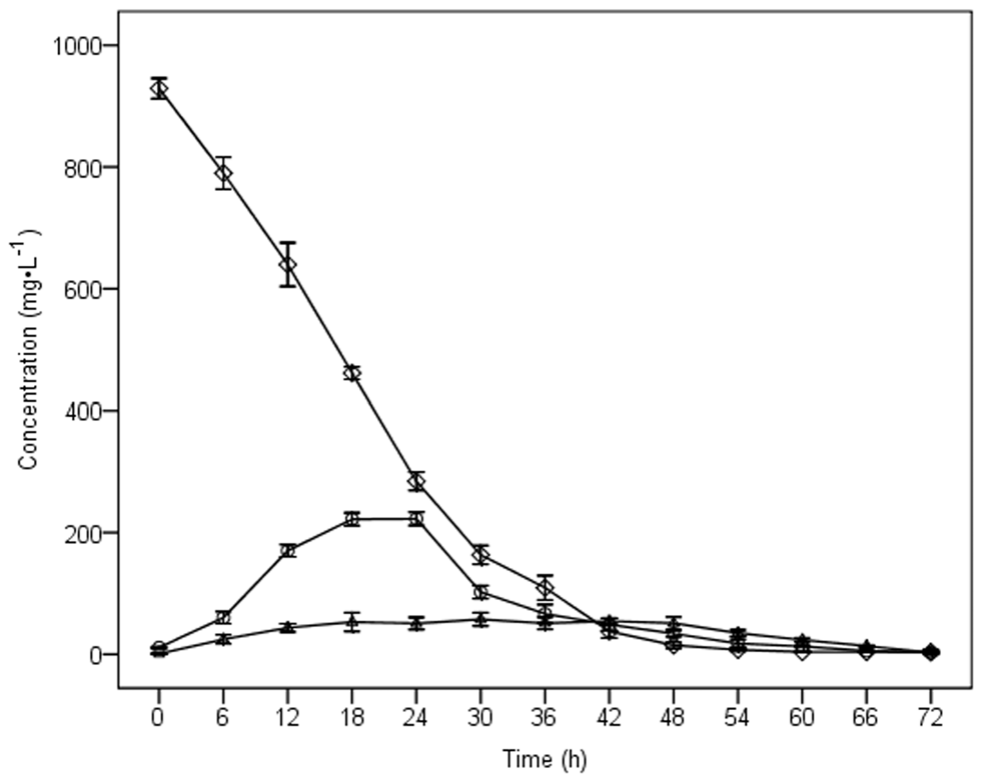
Chemical kinetics of MBC, 2-AB and 2-HB in liquid culture of strain djl-11. ◊, MBC; ○, 2-AB; △, 2-HB. Values are the means of three replicates with standard deviation.

Seed cultures of dj1-11 cells (1% v/v) provided with MBC (1000 mg·L^−1^) as a sole carbon-source degraded approximately 95% of the fungicide in 48 h, with the remaining MBC completely degraded by 72 h (Figure 2). The average degradation rate of MBC by djl-11 was 333.33 mg·L^−1^·d^−1^ in MSM-C_1000_ at 28°C. Varying the concentration of MBC (200–1000 mg·L^−1^) at time of dj1-11 inoculation had no significant effect on overall degradation, with 95% of the fungicide removed after 48 h growth in all treatments (data not shown). No significant MBC degradation was observed in any of the non-inoculated controls, regardless of MBC concentration at time of inoculation.

Two major metabolite peaks were detected during the growth of djl-11 on MBC, these degradation intermediates being identified as 2-aminobenzimidazole (2-AB) and 2-hydroxybenzimidazole (2-HB) by LC and LC-MS using authentic standards.

### Effects of organic substrates, bivalent cations, pH and temperature on MBC degradation

**Figure 3:**
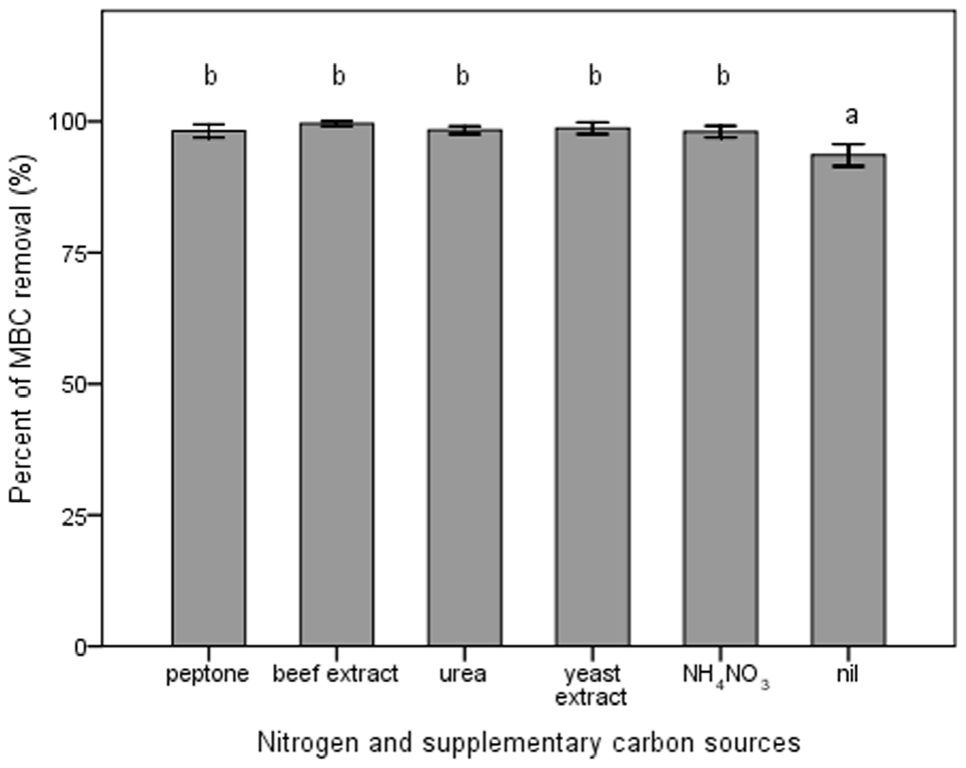
Effects of omitting and substituting NH_4_NO_3_ with organic substrates (N and C sources) on MBC biodegradation. The means of three independent experiments were plotted with error bars indicating standard deviations. Different letters above each column indicate significant differences among treatments (P<0.05).

Strain djl-11 degraded approximately 90% of MBC from MSM-C_1000_ in the absence of NH_4_NO_3_, indicating its capability to utilize MBC as a sole source of carbon and nitrogen (Figure 3). Omission of NH_4_NO_3_ however, resulted in significantly less (P<0.05) MBC degradation compared to that degraded in the presence of this nitrogen-source (Figure 3). Substitution of NH_4_NO_3_ in MSM-C_1000_ with equivalent amounts of organic nitrogen and supplementary carbon sources (*i.e.* peptone, urea, beef extract or yeast extract) had no significant effect on MBC degradation by dj1-11.

**Figure 4:**
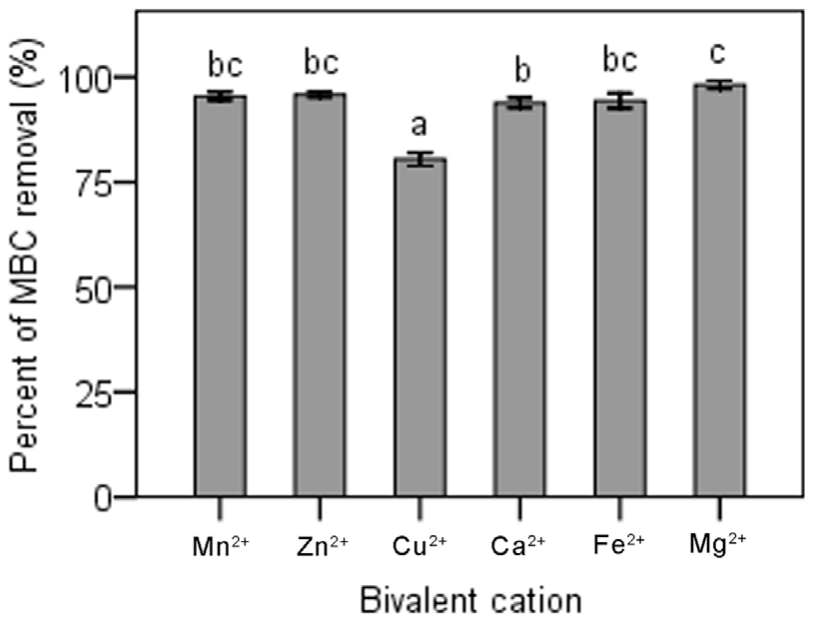
Effects of metal ions on MBC biodegradation. The means of three independent experiments were plotted with error bars indicating standard deviations. Different letters above each column indicate significant differences among treatments (P<0.05).

Substitution of Mg^2+^ in MSM-C_1000_ with equimolar amounts of Mn^2+^, Zn^2+^ or Fe^2+^ had no significant effect on MBC degradation by dj1-11 (Figure 4). In contrast, MBC degradation was significantly decreased (P<0.05) when Mg^2+^ in MSM-C_1000_ was substituted with equimolar Ca^2+^ or Cu^2+^ (Figure 4). Substitution with Cu^2+^ resulted in the lowest overall MBC bio-degradation, significantly less (P<0.05) than that observed in the MSM-C_1000_ containing Mg^2+^ (Figure 4).

**Figure 5:**
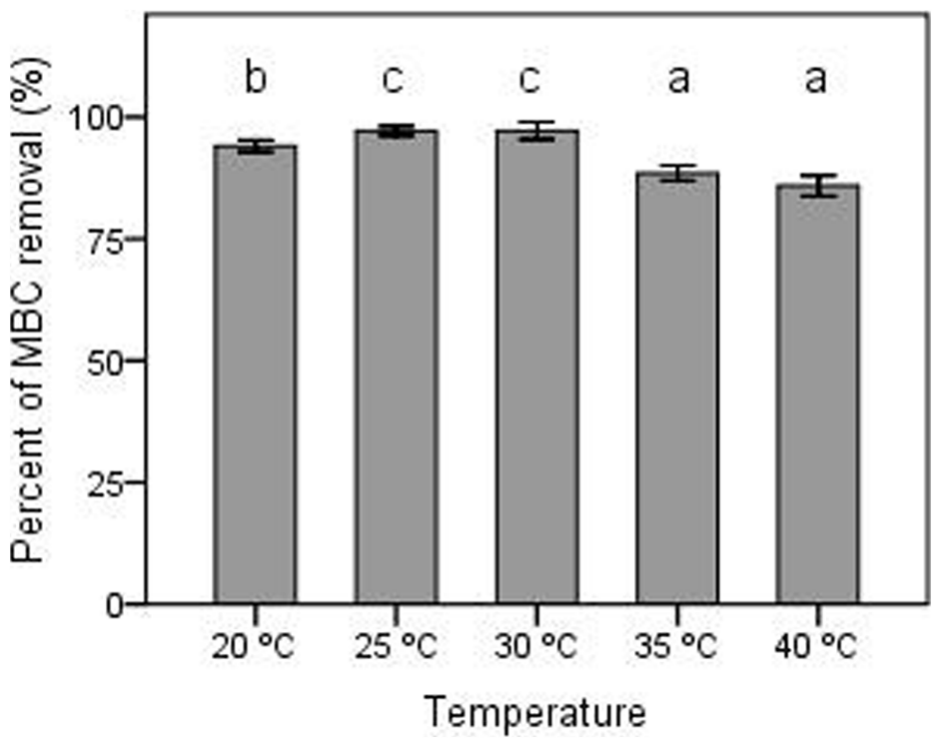
Effect of temperature on MBC biodegradation. The means of three independent experiments were plotted with error bars indicating standard deviations. Different letters above each column indicate significant differences among treatments (P<0.05).

Temperature had a significant (P<0.05) effect on MBC degradation by dj1-11 (Figure 5), with optimal degradation detected in the 25°C to 30°C range. MBC degradation was significantly (P<0.05) lower at 20°C and inhibited further at elevated temperatures of 35°C to 40°C (Figure 5). In contrast, MSM-C_1000_ pH (range pH 4–9) at time of inoculation has no significant effect on biodegradation of MBC by dj1-11 (data not shown).

### MBC-hydrolyzing esterase gene of strain dj1-11

Primers designed from the previously reported MBC-hydrolyzing esterase gene *mheI* amplified a dj1-11 DNA sequence (*Mhe*) consisting of a 729 bp open reading frame starting with the ATG codon, ending with the stop codon TGA and encoding 242 amino acids residues. The predicted amino acid sequence corresponded to a 26.285 kDa protein with an isoelectric point of 6.27. *R. erythropolis* dj1-11 Mhe exhibited 99% amino acid sequence identity with MBC-hydrolyzing esterase encoded by *mheI* from *Nocardioides* sp. strain SG-4G (GenBank accession number GQ454794). The djl-11 *Mhe* DNA sequence of was deposited in GenBank (accession number HQ874282).

## Discussion

At present, only a limited number of bacterial strains capable of degrading MBC have been reported [4], [5], [11]. Strains from the bacterial genus *Rhodococcus* were most often reported, such as *R. erythropolis* [14], *Rhodococcus qingshengii* [15], [16], *Rhodococcus jialingiae* [11]. In this study, strain *R. erythropolis* djl-11 capable of catabolizing and utilizing MBC as the sole carbon and nitrogen sources was isolated. Strain djl-11 showed high MBC-degrading efficacy, with 99% of 1000 mg·L^−1^ MBC being degraded within 72 h. In comparison, *R. qingshengii* djl-6 utilized 100 mg·L^−1^ MBC as the sole carbon source, with an average MBC degradation rate of only 55 mg·L^−1^·d^−1^ [15].

Varying the concentration of MBC (200–1000 mg·L^−1^) at time of inoculation had no significant effect on MBC degradation by strain djl-11, with 95% of the fungicide removed after 48h. In contrast, previous researches on MBC degradation by *Bacillus pumilus* NY97-1 [17] and *Pseudomonas* sp. CBW [5] reported enhanced MBC degradation as concentrations of the fungicide increased. However, MBC concentrations (1–300 mg·L^−1^) in these studies were much lower than those exposed to djl-11, and degradation by *Pseudomonas* sp. CBW was significantly inhibited above MBC concentration of 100 mg·L^−1^ [5].

MBC degradations by *Bacillus pumilus* NY97-1 and *Pseudomonas* sp. CBW were significantly influenced by factors such as pH, temperature and nutrient composition of the culture media [5], [17]. In contrast, MBC degradations by strain djl-11 were not significantly affected by varying the initial pH ranging from 4–9, replacing Mg^2+^ with Mn^2+^, Zn^2+^ or Fe^2+^ or substituting NH_4_NO_3_ with organic substrates (peptone, urea, beef and yeast extracts), the latter providing alternative and additional sources of nitrogen and carbon, respectively. MBC degradation by djl-11 was however, reduced by 5–15% (P<0.05) in the absence of NH_4_NO_3_ when using the fungicide as a sole nitrogen and carbon source or at culture temperature ±5–10°C of the optimum for growth (30°C). Whilst significant, these reductions in djl-11 MBC degrading efficacy are relatively small in comparison with other bacterial strains [5], [17], indicating the robustness of the process by djl-11 and the potential for MBC bioremediation in different environments.

The metabolites 2-AB and 2-HB were identified during the growth of strain djl-11 on MBC, supporting previous studies of MBC catabolism by *Nocardioides* sp. SG-4G [4] and *Pseudomonas* sp. CBW [5]. Whilst MBC degradation by *R. qingshengii djl-6* [15] and *R. jialingiae* djl-6–2 [11] also produced 2-AB, the intermediate benzimidazole (BI) was also detected either in the presence [11] or absence [15] of 2-HB. Notably, BI was not detected during growth of *R. erythropolis* djl-11, *Nocardioides* SG-4G [4] or *Pseudomonas* CBW [5] on MBC. As 2-AB and 2-HB exhibit relatively benign toxicity [18], no attempt was made to define the downstream metabolites. It was proposed that MBC was first converted to 2-AB, which was then transformed to 2-HB, 1,2-diaminobenzene, catechol, and finally to carbon dioxide by *Pseudomonas* sp. CBW [5].

Till now, only one gene, *mheI* from *Nocardioides* sp. SG-4G, which encodes the first enzyme of the pathway that detoxifies MBC by hydrolyzing it to 2-AB was cloned and reported [4]. In this study, MBC-hydrolyzing esterase (Mhe) gene from *R. erythropolis* djl-11 was cloned, and Mhe exhibited 99% amino acid identity to that of *Nocardioides* sp. SG-4G MheI esterase, suggesting that both strains utilize enzymatic hydrolysis as the first step in catabolism and detoxification of MBC to 2-AB. BLAST searches for *Mhe* in all available *Rhodococcus* genomes, including 3 other *R. erythropolis* strains, did not detect any homologous loci with high similarities. The evolutionary origin of Mhe gene and its frequency among other MBC-degrading bacterial strains need to be further elucidated.
